# Non-Pharmacological Interventions Addressing Chronic Pain in People Living with HIV

**DOI:** 10.1007/s11904-025-00734-3

**Published:** 2025-03-14

**Authors:** Yumei O. Chen, Steven A. Safren

**Affiliations:** Department of Psychology, University of Miami, 5665 Ponce De Leon Blvd, Coral Gables, Miami, Florida 33146-2510 USA

**Keywords:** HIV, Chronic pain, CBT, Nonpharmacological treatment, Intervention

## Abstract

**Purpose of Review:**

Chronic pain affects 25–85% of people living with HIV (PLWH), negatively impacting health behaviors and HIV health outcomes. While opioids are frequently prescribed for pain, there are concerns about side effects and addiction potential, and the current consensus guideline advises against their use as a first-line pain management for this population. Therefore, there is an increasing need for non-pharmacological alternatives and adjunctive interventions. This review aims to examine the characteristics, efficacy, and limitations of existing non-pharmacological approaches to chronic pain management in PLWH to inform clinical practices and future research.

**Recent Findings:**

A comprehensive literature search identified 13 clinical trials employing cognitive-behavioral techniques, stress management, positive affect enhancement, and complementary medicine approaches (e.g., yoga, acupuncture, hypnosis). These interventions generally showed significant effects with respect to reducing pain intensity and interference in PLWH, with some also addressing and improving depression, substance use, or antiretroviral medication adherence. However, some were pilot trials and others lacked robust methodologies or sufficient follow-up regarding the ability to definitively determine the durability of these benefits.

**Summary:**

Existing non-pharmacological interventions have potential in addressing pain and related functional impairment in PLWH, such as substance use and emotional well-being. Future research should explore the underlying mechanisms of these interventions and better understand strategies to optimize and establish durability. Incorporating adherence counseling into these interventions could further enhance HIV outcomes by addressing the interconnected challenges of chronic pain and adherence to antiretroviral therapy (ART), thereby supporting both pain management and overall HIV care.

## Introduction

Despite effective control of HIV-related comorbidities and co-infections with antiretroviral therapy, chronic pain continues to be a prominent health concern for people living with HIV (PLWH). Chronic pain, defined as persistent pain lasting more than three months, is associated with adverse health outcomes and impaired physical functioning [1, 2]. The prevalence of chronic pain in PLWH ranges from 25 to 85%, far exceeding the prevalence of 20.9% in the general population [3–7]. Despite this, chronic pain in PLWH has historically been underdiagnosed and undertreated [8], contributing to the unique and multifaceted challenges faced by PLWH.

There are several ways in which HIV can influence pain. Chronic immune activation in HIV increases pro-inflammatory cytokines and chemokines, sensitizing nerves and amplifying pain pathways, thereby heightening the experience of pain [9]. HIV-related neuroinflammation further disrupts normal pain processing pathway and contributes to the development and persistence of chronic pain in PLWH [10]. Additionally, certain ART regimens, such as older nucleoside reverse transcriptase inhibitors (such as stavudine and zidovudine), can have neurotoxic effects and exacerbate neuropathic pain, although newer generations of ART have substantially mitigated this risk [10]. Therefore, PLWH face pain directly related to HIV and sometimes from the treatments designed to manage it.

Chronic pain in PLWH is complex: in addition to the biological factors it is also influenced by psychological, and social factors, as outlined by the biopsychosocial framework [11]. For example, chronic pain caused by persistent immune activation can lead to psychological distress, which can compound the experience of pain and lead to more distress, perpetuating the vicious cycle [12]. Additionally, psychosocial factors like adverse childhood experiences, mental illness, and reduced social support may exacerbate psychological distress and negatively impact health behaviors, such as missed clinic visits and poorer adherence to ART, leading to worsened chronic pain and other adverse clinical outcomes in PLWH [13–16]. Therefore, both medical/pharmacological and psychosocial interventions for pain for PLWH should be explored and studied.

Accordingly, chronic pain management for PLWH currently involves both pharmacological and non-pharmacological approaches, but opioid analgesics are not recommended as a first-line treatment due to their risks and potential dependency [17, 18]. One issue is that HIV-associated neuropathic pain is often resistant to traditional analgesics like nonsteroidal anti-inflammatory drugs (NSAIDs) or opioids [19, 20]. The current guidelines released by the Infectious Diseases Society of America (IDSA) in 2017 [17] emphasize nonopioid options, such as early initiation of ART, gabapentin, capsaicin, and medical marijuana, which have shown efficacy for HIV-associated sensory neuropathy but come with limitations [21].

Non-pharmacological interventions, such as cognitive behavioral therapy (CBT), offer several advantages for PLWH when used alone or alongside medications. The 2017 IDSA guidelines recommended CBT as a first-line approach for managing chronic pain in PLWH [17], citing its minimal side effects and potential to alleviate the burden of extensive medication regimens. These interventions empower patients with practical tools for self-management, which can enhance ART adherence, retention in care, and overall well-being [22–24]. Additionally, these types of non-pharmacological interventions often address co-occurring conditions such as depression and substance use [11].

Despite these advances, there remains a notable lack of psychological and behavioral pain management strategies tailored for PLWH [7, 17]. To address this gap in the literature, the present review examined the emerging body of research that assesses the efficacy of non-pharmacological interventions for chronic pain in PLWH and identifies opportunities for future research and development of targeted interventions. Importantly, these approaches are not intended to replace pharmacological management when warranted, especially in cases of severe pain. Instead, non-pharmacological interventions can complement medication, offering a more holistic approach to pain management.

## Methods

### Literature Search Strategy

A comprehensive literature search was conducted to identify relevant studies examining non-pharmacological interventions for chronic pain in PLWH. Electronic databases, including PubMed/MEDLINE, PyscINFO, and Cochrane Library, were searched for publications from January 2000 to December 2024. Keywords in the search included “HIV,” “chronic pain,” “pain,” “neuropathy,” “CBT,” and “non-pharmacological.” Boolean operators “AND” and “OR” were employed to refine search results and capture relevant studies. Reference lists of included studies and relevant review articles were hand-searched to identify additional articles not captured in the initial search. A total of 17 studies were summarized in the Results and Discussion sections.

### Data Extraction and Synthesis

Data extraction was performed independently by the first author using a standardized form. Extracted data included study characteristics (e.g., author, year of publication), participant characteristics (e.g., sample size, demographics), intervention details (e.g., type, duration), and key findings related to the efficacy and feasibility of the treatment. Studies were psychosocial and complementary, including traditional CBT interventions and those with CBT components but also mindfulness practices, positive affect training, or social support facilitation.

## Results

### Psychosocial Interventions

The literature review found 10 published clinical trials examining psychosocial interventions for PLWH (see Table 1). Nine of these are randomized controlled trials (RCT), and one is a single-arm trial, all but one with pain as the primary outcome.

**Table 1:** Design, methodology, and outcomes of reviewed studies

Authors (year)	Population (*N*)	Inclusion criteria	Intervention	Comparison arm (s)	Findings
**Psychosocial Interventions: Randomized Controlled Trial (RCT) Studies** (*N* = **5)**					
Jones et al. [2024, 30]	Adults living with HIV (*N* = 278)	Reported at least moderate pain for three months or more	*STOMP*: 12 sessions with pain education, self-management strategies and peer support	Enhanced usual care comparison	STOMP was associated with a significant reduction in pain-related outcomes post-intervention and at the three-month follow-up
Uebelacker et al. [2023, 31]	Adults living with HIV (*N* = 187)	Reported chronic pain with elevated pain severity ratings and depression symptoms	*HIV-PASS*: 7 sessions with behavioral activation (BA), self-management strategies, and medication adherence counseling	Health education comparison	HIV-PASS was associated with significantly lower pain-related interference with daily activities immediately after intervention period
Addington et al. [2020, 41]	Adults newly diagnosed with HIV (*N* = 159)	Diagnosed with HIV in the past 12 weeks and reported pain in the past month	*IRISS*: 5 sessions with 8 positive affect skills (pain secondary outcome)	Supportive personal interview	The intervention arm reported increased frequency of musculoskeletal pain but no significant changes in pain interference
Parker et al. [2016, 27]	amaXhosa women living with HIV (*N* = 27)	Reported pain beyond typical everyday pains	6 weekly sessions led by peers with exercise and education program	Health education comparison	Both arms experienced similar pain reduction, with workbooks alone proving as effective as the full intervention
Evans et al. [2003, 25]	Adults living with HIV (*N* = 61)	A diagnosis of HIV-related peripheral neuropathy with moderate to severe rating	6 sessions with cognitive and behavioral techniques	Supportive psychotherapy	The intervention arm showed greater reductions in pain-related outcomes though not significant
**Psychosocial Interventions: Feasibility and Pilot RCT Studies** (*N* = **5)**					
Scott et al. [2021, 36]	Adults living with HIV (*N* = 38)	Neuropathic pain that lasted for more than 3 months with elevated pain intensity and interference and moderate depression symptoms	*ACT OPEN*: 12 sessions with educational videos about pain and guided exercises to improve psychological flexibility	Waitlist comparison	The intervention arm showed small to moderate improvements in pain-related outcomes and depression symptoms
Palfai et al. [2020, 32]	Adults living with HIV (*N* = 8)	Reported chronic pain that was on average moderate or greater over the past week and heavy drinking in the past month	7 sessions with BA and self-management strategies	NA	The intervention demonstrated good feasibility, high acceptability, and preliminary efficacy in reducing pain and heavy drinking
Moore et al. [2019, 33]	Older adults living with HIV (*N* = 55)	50 years or older who reported chronic pain and at-risk substance use by NIH guidelines	*CBT/TC/TXT*: 8 sessions combining CBT, Tai Chi, and Motivational Interviewing-reinforced text messaging	Routine support arm and assessment only arm	The intervention arm reported the greatest reductions in heavy drinking, pain, and improvement in physical functioning
George et al. [2017, 39]	Adults living with HIV (*N* = 32)	Reported neuropathic and/or musculoskeletal pain for at least 3 months	8 group sessions with mindfulness-based stress reduction techniques	Education comparison	The intervention arm reported modest improvements in pain-related outcomes and perceived stress though not significant, more durable effects
Trafton et al. [2012, 26]	Adults living with HIV (*N* = 70)	Reported chronic pain	*CBTPM*: 12 group sessions focused on various cognitive-behavioral techniques	NA	The intervention arm showed significant improvements in pain-related outcomes and physical functioning and reduction in psychological symptoms
**Complementary and Alternative Interventions: RCT Studies** (*N* = **2)**					
Mawar et al. [2015, 44]	Adults living with HIV (*N* = 61)	CD4 count above 400 cell/µl and functional impairment within the normal range	12 sessions of Sudarshan Kriya yoga practice guided by a yoga instructor	Standard care	The intervention arm reported a significant improvement in overall quality of life scores and in 3 specific domains: physical, psychological, and level of independence
Shiflett & Schwartz. [2010, 46]	Men aged 13 years or older living with HIV (*N* = 125)	Reported HIV-related lower extremity peripheral neuropathy	Twice weekly for 6 weeks and once weekly for 8 weeks - amitriptyline hydrochloride and standardized acupuncture regimen	Three comparison arms: Acupuncture only Amitriptyline only Placebo	Acupuncture showed a steady decline in pain intensity over the treatment period while amitriptyline showed a diminished effect; and acupuncture was associated with higher pain relief with amitriptyline not present
**Complementary and Alternative Interventions: Feasibility Study** (*N* = **1)**					
Dorfman et al. [2013, 45]	Adults living with HIV (*N* = 36)	Reported HIV-related neuropathic pain and were on a stable pain treatment regimen	3 sessions of self-hypnosis training with techniques that help manage pain and improve quality of life	NA	Participants reported a significant reduction in pain and improvement in overall quality of life, and the pain reduction was stable for 7 weeks post intervention

Note. Pain−related outcomes include pain intensity and pain interference

The first published trial of CBT for HIV-related pain was conducted by Evans and colleagues [25]. This study was a two-arm RCT (*N* = 61) with supportive psychotherapy as the comparison arm. The intervention entailed six weekly sessions focused on cognitive and behavioral strategies to help participants manage their pain and emotional distress, such as depression and anxiety. While the intervention arm showed greater reductions in pain intensity and interference, the differences were not statistically significant. Additionally, a higher dropout rate in the intervention arm emerged, and the authors posited that some participants found the treatment overly complex or burdensome. These findings highlight the challenges of developing and tailoring effective interventions during an earlier phase of CBT’s application to chronic pain, emphasizing the need for balancing therapeutic rigor with participant preferences.

Building on this evidence, Trafton and colleagues [26] tested a program focusing on specific CBT techniques for managing chronic pain, such as cognitive restructuring to reduce maladaptive thinking patterns, cognitive reconceptualization to change how individuals perceive pain, and exercise and sleep management. The trial was a single-arm study called CBT-Based Pain Management (CBTPM) implemented in three public primary care clinics. Participants (*N* = 70) with moderate to severe pain lasting more than 3 months were enrolled. CBTPM was associated with large effect size improvements on pain intensity, interference, pain-related anxiety, and pain acceptance at all time points across all three clinics. Therefore, integrating CBTPM into primary HIV care was deemed feasible, acceptable, and potentially effective in pain management for PLWH.

To implement CBT-based pain interventions in broader settings, Parker and colleagues [27] tested a 6-week, two-arm randomized controlled trial of a peer-led program tailored for amaXhosa women living with HIV in the community of Khayelitsha, South Africa (*N* = 27). Participants in the intervention arm received educational workbooks on problem-solving and goal-setting and progressive aerobic and strengthening exercises. Those assigned to the comparison arm received educational workbooks alone. Results indicated that both arms achieved comparable pain reduction from baseline when they received routine care, suggesting that workbook use alone was as effective as the intervention program. Therefore, depending on the specific needs of the communities, providers can decide between workbook alone and a peer-led model in clinical implementation of this approach.

Skills to Manage Pain (STOMP) intervention is grounded in social cognitive theory (SCT), which shares foundational principles with CBT, such as emphasis on self-regulation strategies. SCT posits that even in the face of stressors like pain, people can adapt and change health behaviors through internal motivations and outcome expectations [28]. This intervention was found to be both feasible and cost-effective in a pilot study [*N* = 44; 29] and was then tested in a two-arm RCT [*N* = 278; 30]. STOMP included 12 weekly group and one-on-one sessions focusing on pain education, pain self-management behaviors (e.g., physical activities and your pain, losing weight to improve your pain, relaxation skills to prevent your pain, sleeping better to help your pain, and thinking different about your pain), and peer support to learn self-management skills from other PLWH. Results indicated that STOMP was associated with a statistically significant arm difference in pain ratings post-intervention and at the three-month follow-up. These findings support the efficacy of STOMP as an intervention for chronic pain in PLWH.

The HIV-Pain and Sadness Support (HIV-PASS) intervention [31] is grounded in the principle that pain and depression are linked and that depression can exacerbate the experience of pain. In a two-arm RCT, 187 PLWH experiencing both pain and depressive symptoms were enrolled and randomly assigned to either the HIV-PASS intervention or a time- and attention-matched health education comparison arm. The three-month HIV-PASS program included CBT techniques such as cognitive restructuring to address catastrophic beliefs about pain, behavioral activation strategies to identify meaningful life goals and engage in activities that bring them joy and fulfillment, time-based pacing techniques, and medication adherence counseling to promote self-management. Results showed that compared to health education comparison, HIV-PASS was associated with significantly lower pain-related interference with daily activities at the end of month 3, though this difference diminished at the 12-month follow-up.

CBT-based interventions can also effectively address maladaptive coping behaviors for chronic pain, such as substance use. In a single-arm proof-of-concept study targeting chronic pain and heavy alcohol drinking [32], participants (*N* = 8) first completed an in-person session with a licensed clinical psychologist who utilized motivational interviewing (MI) strategies to help the participants understand how heavy drinking influenced their experience of pain and articulate their reasons and motivation for change. Participants then engaged in six videoconferencing sessions focused on self-management strategies, including activity pacing, harm reduction for drinking, and sleep hygiene. Results demonstrated good feasibility, high acceptability, and a preliminary effect on reducing pain and heavy drinking.

Similarly, a chronic pain and substance use intervention that integrated CBT, tai chi, and MI-reinforced text messaging (CBT/TC/TXT) was evaluated in a three-arm RCT [*N* = 55; 33]. Participants in the intervention arm attended weekly group sessions to learn CBT strategies, such as cognitive restructuring, to cope with negative thoughts and emotions and understand and change problematic patterns of substance use. Following the CBT session, they practiced one-hour Yang-style Tai Chi and received weekly text reminders of session content and home practice. The CBT/TC/TXT group showed statistically significant reductions in heavy drinking, pain, and improvement in physical performance, compared to the routine support arm and assessment-only arm. Additionally, a high completion rate (84% attended the 12-week program, and > 60% attended at least 6 of 8 sessions) was recorded, and qualitative data highlighted that participants rated the sessions as extremely useful and enjoyable.

The ACT OPEN intervention adopted principles of acceptance and commitment therapy (ACT), a behavioral approach under the umbrella of CBT that focuses on fostering psychological flexibility so individuals can experience pain with openness, develop present-moment awareness, and engage in value-driven activities [34, 35]. This online intervention tailored for PLWH with neuropathic pain was developed and piloted in a two-arm RCT [*N* = 38; 36]. The intervention featured quotations from PLWH living with chronic pain, informative videos about pain, guided exercises such as metaphors, mindfulness practices, experiential exercises, value clarification, and goal setting. While results showed small to moderate improvements in pain intensity, pain interference and depression, significance testing was limited by sample size. Participants rated treatment credibility and satisfaction on par with prior CBT-based pain interventions, supporting its potential for further evaluation in a full-scale RCT.

Mindfulness-Based Stress Reduction (MBSR) is another appealing option consistent with the principles of CBT, and it can be delivered in a group format and has been proven to be beneficial in affecting various HIV-related health outcomes [37, 38]. In a two-arm RCT (*N* = 32) evaluating an 8-week MBSR program [39], those in the intervention condition engaged in weekly mindfulness practices, including breathing techniques, body awareness meditation, and yoga. The comparison arm participated in weekly discussions on HIV-associated pain without structured mindfulness exercises. Results showed modest improvements in pain measures and perceived stress with no significant arm differences. However, at the three-month follow-up, 80% of the MBSR participants reported still practicing MBSR and improved pain intensity, whereas those in the comparison arm reported worsened pain intensity (4% greater than the baseline).

Positive affect therapy is another evidence-based approach that similarly promotes psychological flexibility, resilience, and mindfulness and has been used for chronic pain management [40]. In a two-arm RCT with adults newly diagnosed with HIV [*N* = 159; 41], participants in the intervention arm completed five weekly sessions on eight positive affect skills (e.g., gratitude, mindfulness, and positive reappraisal), while the control arm participated in supportive personal interviews. Although the study was not designed specifically to address pain, a secondary analysis of pain-related outcomes revealed notable findings. At post-intervention and 10-month follow-up, the intervention arm reported significantly less headache-related interference than the comparison arm. The intervention arm also reported significantly higher frequency of musculoskeletal pain but not levels of pain interference, although this difference in frequency and interference diminished at the final assessment. Because the study was not designed prospectively for pain as the primary outcome, these results should be interpreted with caution. The authors suggested that positive affect therapy improved participants’ ability to manage pain, reducing its impact on daily activities, though the initial, but not sustained, increased musculoskeletal pain may warrant further investigation.

### Complementary and Alternative Interventions

In addition to psychosocial approaches, complementary and alternative treatment modalities are recommended as non-pharmacological treatments for chronic pain in PLWH [17, 42]. The current review found two RCTs and one single-arm study (see Table 1).

Yoga is considered beneficial for PLWH based on a meta-analysis [43] and has been assessed for pain management. In an open-label RCT [*N* = 61; 44], PLWH were randomized to either yoga or standard care. The yoga group practiced yoga once weekly for 12 weeks, and six domains of health-related quality of life were assessed (e.g., pain, energy, sleep). Results showed a 6% improvement in overall quality of life and a 12% improvement in the physical health domain, although the specific contribution of pain relief could not be isolated.

Hypnosis is also commonly utilized for pain and assessed by Dorfman and colleagues [45] on HIV-related neuropathic pain. In a single-arm study, 36 PLWH received three weekly training sessions in self-hypnosis and reported a significant reduction in pain ratings and an increase in measures of affect and quality of life from baseline. The improvements were found to last for at least 7 weeks post-intervention. The authors concluded that although brief hypnosis interventions hold promise for managing neuropathic pain in PLWH, individual variations in susceptibility to hypnosis can affect the treatment outcomes.

Lastly, acupuncture can also be considered a viable treatment for chronic pain in PLWH, especially appealing to individuals seeking alternatives to medications. In a 2 × 2 crossed-design RCT involving acupuncture and amitriptyline and their corresponding comparison arm, sham acupuncture, and placebo pill [*N* = 125; 46], both acupuncture and amitriptyline showed significant main effects on pain intensity and significant interaction. Specifically, it was found that when amitriptyline was not present, acupuncture was associated with a higher degree of pain relief. Therefore, acupuncture alone, or used in conjunction with other treatments, shows promise in managing HIV-related neuropathic pain effectively.

## Discussion

To our knowledge, this paper is the first published work to review non-pharmacological interventions for chronic pain and its associated comorbidities within PLWH. The reviewed studies highlight the potential of both psychosocial and complementary and alternative interventions for managing chronic pain and co-occurring issues such as depression and substance use in PLWH. While some studies [32, 36] lacked the statistical power to detect significant outcomes due to small sample sizes, most demonstrated statistically significant treatment effects on pain-related outcomes. Although a few studies [27, 39] failed to show significant differences between intervention and control arms, it is worth noting that control participants often received health education with therapeutic elements. Overall, these interventions reflect a shift toward holistic pain management modeled after the biopsychosocial framework, leveraging participants’ strengths, self-efficacy, and social networks to promote resilience and adaptive coping.

Grounded in the biopsychosocial framework of chronic pain [11], almost all of the treatments employed multimodal approaches to target the multifaceted nature of pain and comorbid conditions. These interventions incorporated a range of strategies, including text messaging for ongoing support and reminders, mindfulness-based practices to manage emotional distress and pain catastrophizing, and physical exercises to improve mobility and overall functionality. By addressing both psychological and physical dimensions of pain, these approaches sought to improve pain-related outcomes and overall quality of life.

Several studies [26, 30, 39] utilized a group-based intervention format to foster a supportive environment for participants to share pain experiences. This group dynamic helped reduce feelings of isolation and allowed participants to learn adaptive coping strategies from each other. Hearing others’ stories can also validate individuals’ feelings and experiences and reduce feelings of frustration or helplessness. A recent meta-analysis [47] highlighted how group psychotherapy could effectively reduce pain intensity and improve adjacent issues. These findings highlight the value of multimodal and group-based approaches in addressing the multifaceted challenges of pain within PLWH.

Several studies also leveraged participants’ existing self-management skills developed through successfully managing HIV to enhance pain-related outcomes. Self-management refers to an individual’s control of their behavior, particularly regarding the pursuit of a specific objective [48]. For PLWH, self-management involves adhering to medication regimens, making lifestyle adjustments, and navigating healthcare systems. For example, interventionists in HIV-PASS [31] focused on setting goals aligned with participants’ prior successes to ensure that patients feel confident in their ability to overcome pain-related obstacles. Similarly, the STOMP intervention [30] encouraged participants to engage in problem-solving and clinical decision-making to set and achieve personal goals related to pain management. By tapping into the existing self-efficacy cultivated through years of managing HIV, individuals may feel empowered and ultimately achieve improved quality of life and overall well-being.

While these interventions effectively build on existing self-management skills, researchers should recognize that simultaneously managing two chronic conditions can create additional stressors and competing priorities [49]. Individuals may find themselves juggling multiple medical appointments, medical regimens, and treatment plans, all while contending with the physical, emotional, and social ramifications of chronic pain and HIV. This compounded burden may hinder the ability to consistently apply self-management skills acquired in treatment, as seen in one study [31] where the treatment effects did not persist at follow-up. Therefore, ongoing support and reinforcement are essential to help participants sustain and apply learned skills beyond the treatment period.

The five pilot/feasibility studies share several limitations that may affect the reliability and validity of their findings. While smaller sample sizes are typical of pilot studies, careful interpretation of the findings is necessary due to the potential for effect size inflation [50]. A high attrition rate with unclear reasons was observed in one study [25], which may further reduce statistical power and generalizability. Additionally, two studies [32, 45] utilized a single-arm design, limiting the ability to draw conclusions about the efficacy of the intervention. To address these issues, future pilot studies should conduct sensitivity analyses to evaluate the robustness of results and implement feasibility measures beyond total attrition rates, such as qualitative interviews on barriers and facilitators to study visits [51]. Understanding these factors can provide actionable insights into improving study design and participant retention.

Moreover, most studies reviewed in this paper specifically recruited PLWH living with neuropathic pain, highlighting the need for future studies to examine how interventions may vary in their effectiveness based on pain type. Chronic pain can manifest as nociceptive or neuropathic, and they differ significantly in their physiological mechanisms and responses to treatments [7]. Future studies should investigate whether interventions are equally effective across these types of pain or if tailored approaches are necessary to address their unique characteristics. For example, psychosocial interventions may more effectively address the emotional and psychological distress associated with neuropathic pain, while physical activities might improve functionality and reduce the severity of nociceptive pain. By tailoring the interventions to the specific type of pain, providers can address individuals’ specific needs and optimize treatment outcomes.

## Conclusion

In conclusion, the challenges faced by PLWH and chronic pain highlight the importance of addressing the multifaceted nature of their conditions. In general, psychosocial approaches appear to be beneficial in improving pain-related outcomes in PLWH who experience pain. Complementary and alternative interventions such as yoga, hypnosis, and acupuncture also demonstrated potential but may need more rigorous/randomized trials to have evidence for efficacy. Through this comprehensive review, healthcare providers can hopefully gain insight into the breadth of non-pharmacologic treatments available for PLWH and make informed decisions in selecting treatments that align with individual needs and preferences.

## Key References

- Slawek DE. People living with HIV and the emerging field of chronic pain—what is known about epidemiology, etiology, and management. Curr HIV/AIDS Rep. 2021;18:436–42.
-
  - The paper reviews the current understanding of chronic pain in PLWH and specifically summarizes knowledge on the etiology, epidemiology, and management of chronic pain in this population.
- Bruce RD, Merlin J, Lum PJ, Ahmed E, Alexander C, Corbett AH, et al. 2017 HIVMA of IDSA clinical practice guideline for the management of chronic pain in patients living with HIV. Clin Infect Dis Off Publ Infect Dis Soc Am. 2017;65:e1–37.
-
  - The paper providers a comprehensive guideline and recommendations for healthcare providers treating chronic pain in PLWH.
- Uebelacker LA, Pinkston MM, Busch AM, Baker JV, Anderson B, Caviness CM, et al. HIV-PASS (pain and sadness support): randomized controlled trial of a behavioral health intervention for interference due to pain in people living with HIV, chronic pain, and depression. Psychosom Med. 2023;85:250.
-
  - A randomized controlled trial that demonstrated the effectiveness of a behavioral health intervention for PLWH who also experience chronic pain and depression.
- Scott W, Guildford BJ, Badenoch J, Driscoll E, Chilcot J, Norton S, et al. Feasibility randomized-controlled trial of online acceptance and commitment therapy for painful peripheral neuropathy in people living with HIV: The OPEN study. Eur J Pain Lond Engl. 2021;25:1493–507.
-
  - A randomized controlled trial that demonstrated the feasibility and acceptability of an online ACT-based therapy for chronic pain in PLWH.

## Data Availability

No datasets were generated or analysed during the current study.
